# Pediatric multi-drug resistant-tuberculosis and HIV co-infection in a resource—limited setting: a case report

**DOI:** 10.1186/s13104-018-3148-5

**Published:** 2018-01-22

**Authors:** Christelle Géneviève Jouego, Valirie Ndip Agbor, Juergen Noeske, Ndo Akono Manuel, Leo Njock Ayuk

**Affiliations:** 1 Tuberculosis Reference Laboratory Bamenda, P.O Box 586, Bamenda, Northwest Region Cameroon; 2 Ibal Sub-divisional Hospital, Oku, Northwest Region Cameroon; 3 Consultant at the National Tuberculosis Program, Yaoundé, Cameroon; 4 Bamenda Regional Hospital, Northwest Region, Bamenda, Cameroon

**Keywords:** Contact tracing, Pediatric multi-drug resistant tuberculosis, Xpert MTB/RIF, Case report, Cameroon

## Abstract

**Background:**

Tuberculosis remains a major cause of morbidity and mortality worldwide, especially in developing countries. The diagnosis and treatment of multi-drug resistant tuberculosis (MDR-TB) in children remain a major limitation in this setting, largely due to difficulties in isolating *Mycobacterium tuberculosis* from pediatric specimens, management with toxic second line drugs, and practically the inexistence of contact tracing. In 2016, the World Health Organization (WHO) recommended a standardized 9-month regimen for adults and children in zones which are highly endemic for the human immunodeficiency virus (HIV). Herein, we present a case of pediatric MDR-TB/HIV co-infection highlighting the difficulties in treatment and the importance of contact tracing.

**Case presentation:**

A 6-year old male infant from the West Region of Cameroon infected with HIV who presented at a local health center with a 10 days history of productive cough associated with nocturnal fever and abdominal pains non responsive to broad spectrum antibiotics. A sputum sample analysis requested was smear positive for acid-fast bacilli, and he was initiated on quadritherapy for drug sensitive pulmonary tuberculosis. Since he was a household contact of the mother who was being managed in a referral hospital for MDR-TB at 1 month of treatment, and given his critical clinical situation, a gastric aspirate was repeated and sent for Xpert MTB/RIF to the Tuberculosis Reference Laboratory which was positive for a Rifampicin resistant strain of *M. tuberculosis.* The short 9 months regimen against MDR-TB was then initiated. During the course of his management, he developed minor side effects of the drugs which were managed symptomatically.

**Conclusion:**

Even though pediatric MDR-TB is difficult to confirm, it can be treated with favorable clinical outcomes using the short regimen recommended by the WHO. Experts involved in the control of tuberculosis over the national territory should consider adopting routine contact tracing for all cases of tuberculosis particularly amongst children.

## Background

Multi-Drug Resistant Tuberculosis (MDR-TB) is a form of drug resistant tuberculosis (TB) in which the etiologic agent *Mycobacterium tuberculosis* can no longer be killed by at least the two best antibiotics, Isoniazid (INH) and Rifampicin (RIF), commonly used in the treatment of TB. As a result, this form of the disease is difficult to treat, and requires longer periods, from 9 to 12 months, of treatment. According to the global TB report in 2015, it is estimated that 25,000 children developed MDR-TB worldwide [1]. Pediatric MDR-TB remains a public health challenge of growing concern, accounting for an estimated 15% of all global cases of MDR Tuberculosis [2], with higher rates in developing countries. Since the highest incidence of TB and prevalence of Human Immunodeficiency Virus (HIV) are recorded in Sub-Saharan Africa, children living in this region bear the greatest burden of TB/HIV co-infection. However, a search of MEDLINE through PubMed revealed only two reported cases on pediatric MDR-TB in Nigeria in 2012 [3] and Egypt in 2016 [4]. A lack of adequate diagnostic tools for TB in most resource-limited settings is more likely responsible for under diagnosis and underreporting of most cases of MDR-TB, especially in the pediatric population. This is due to the fact that most diagnosis among children is subjective thus challenging to confirm, and the treatment side effects are usually difficult to manage [5].

Although children experience fewer adverse effects to second line anti-TB drugs the outcomes in this population can be at least as good as those reported for adults [6]. Herein, we present a case of pediatric MDR-TB coinfected with HIV in a 6-year-old male, with emphasis on contact tracing and outcome on the 9 month regimen recommended by the WHO.

## Case presentation

A 6-year-old male from the West Region of Cameroon diagnosed with HIV 3 years back and on pediatric first line highly active antiretroviral therapy (HAART) regimen—Abacavir, Lamivudine and Efavirenz (ABC/3TC/EFZ),—was referred from a health center to our institution for suspicion of pulmonary TB based on a 10 days history of productive cough, intermittent nocturnal fever and generalized abdominal pain, which persisted despite administration of undocumented broad spectrum intravenous antibiotics for 8 days. His mother was on the first month of hospitalization at our institution for management of MDR-TB, a referral hospital for MDR-TB.

On examination, the child had respiratory distress with a pulse rate of 137 beats/min, respiratory rate of 22 cycles/min and temperature of 40.1 °C. His weight and height were 20 kg and 80 cm, respectively. There was pallor of the conjunctivae, palms and soles. The BCG scar was present on the left forearm. The heart was tachycardic with audible functional systolic murmurs. A bilateral consolidation syndrome was also noted on examination of the lungs. An initial diagnosis of pediatric pulmonary TB/HIV coinfection, associated with a clinical anaemia was made. MDR-TB was the principal differential diagnosis.

**Fig. 1:**
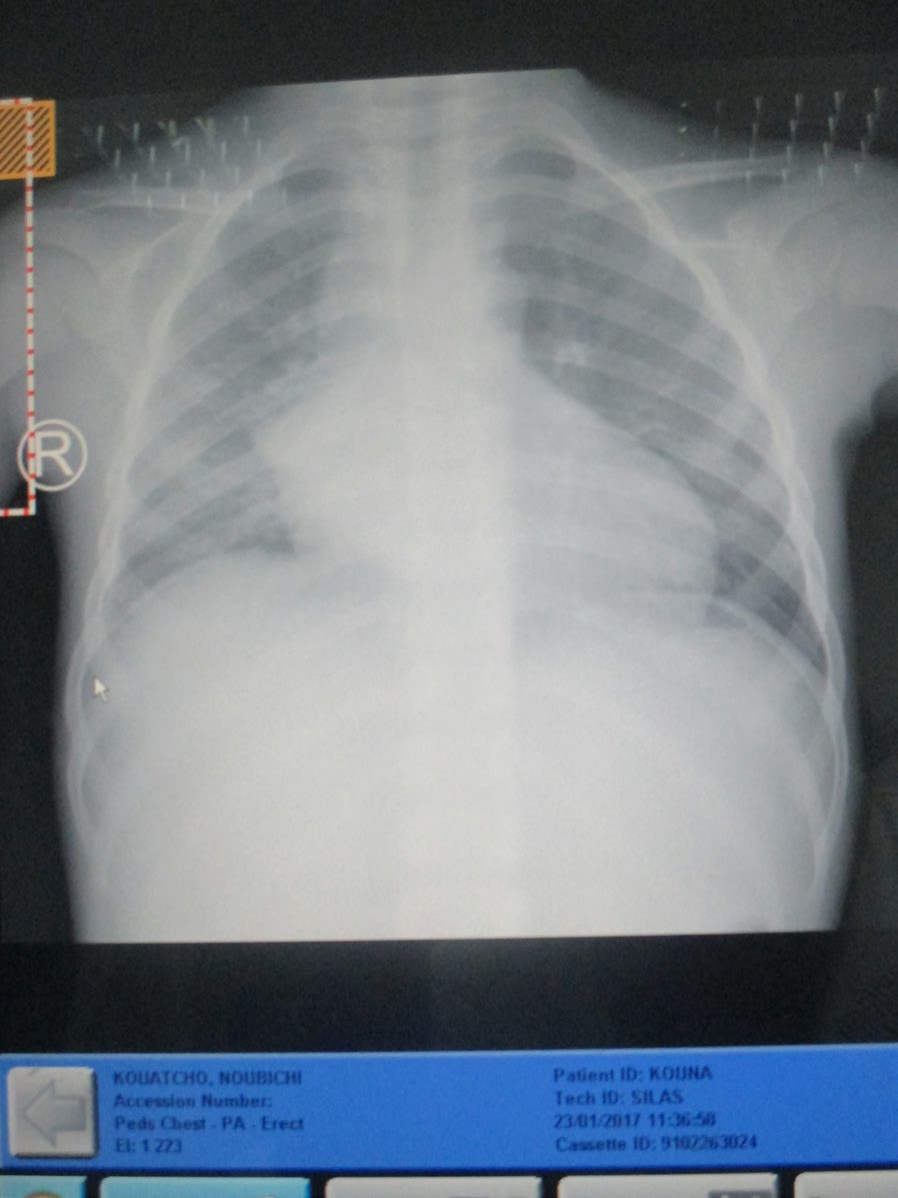
An antero-posterior view chest X-ray revealed a bilateral micro-nodular interstitial opacity associated with bilateral hilar adenopathy

A sputum analysis done was positive for acid-fast bacilli. Since he was a household contact of the mother and given that he became very ill, and unable to produce one more sputum, we chose to collect the gastric aspirate and sent for gene Xpert MTB/RIF and culture/DST in other to make a good diagnosis early enough. The gastric aspirate was positive for a *Mycobacterium tuberculosis* Rifampicin resistant strain using Xpert MTB/RIF assay. After performing the proportional method -drug susceptibility testing (PM-DST) on Lowenstein Jensen slopes, the child was found to have the same resistance pattern like the mother (Isoniazid-susceptible, Rifampicin-resistant, Ethambutol-susceptible, Kanamycine-susceptible, Ofloxacine-susceptible and streptomycine-not done). Due to the severe anaemia at 6.1 g/dl, he received 20 ml/kg (400 ml) of whole blood in emergency. The results of his pre-therapeutic work-up for MDR-TB regimen were as follows: slightly raised Aspartate Aminotransferase but normal Alanine Aminotransferase at 68 and 28.5 U/l, respectively; Serum Creatinine was normal at 0.45 mg/dl. An antero-posterior view chest X-ray revealed a bilateral micro-nodular interstitial opacity associated with bilateral hilar adenopathy (Fig. 1). Also an audiogram and electrocardiogram (ECG) done were not contributive.

After discussing the case with the MDR-TB consultant of the National Tuberculosis Program (NTP), with regards to the results of his pre-therapeutic work-up, the short 9 month—regimen was initiated according to his weight: 4 months of Amikacin/Moxifloxacin/Prothionamide/Isoniazid/Clofazimin/Ethambutol/Pyrazinamid for the initiation phase, followed with 5 months of Moxifloxacin/Clofazimin/Ethambutol/Pyrazinamid for the continuation phase [4(AmMfxPtoHCfzEZ)/5(MfxCfzEZ)]. His viral load was 23,144 copies/µl and the CD4 cell count 149cells/mm.

Day 36 of hospitalization was marked by another episode of decompensated severe anaemia at 3.9 g/dl and non-infectious enteritis (with abdominal pains and watery stool). This prompted another transfusion of whole blood cells at 20 ml/kg (400 ml), and iron and folic acid syrup at a dose of 6 mg/kg/day (120 mg) were continued twice daily for a month. In addition, the patient presented with mild bilateral knee pain which was immediately controlled with 2 mg/kg (40 mg) dose of diclofenac divided twice a day. He also presented with a progressive brownish discoloration of the eyes with no visual impairment for which he is been followed-up. During the course of the treatment, the child was doing well and was able to produce a good sputum sample; he was discharged 4 months after the initiation phase with consecutively negative monthly sputum controls (Table 1). The child is presently on the ninth month of treatment with no further complaints.

**Table 1:** Monthly sputum control results of the child

Sputum sample control	Date	Smear microscopy with LED fluorescence microscopy	Xpert MTB/RIF	Culture (MGIT and LJ)
Month 0	05/12/2016	1+	MTB detected and RIF detected	TBC
Month 1	17/01/2017	No AFB seen		Negative
Month 2	09/02/2017	No AFB seen		Negative
Month 3	10/03/2017	5 AFB seen		Negative
Month 4	04/04/2017	No AFB seen		Negative
Month 5	08/06/2017	No AFB seen		Negative
Month 6	05/07/2017	No AFB seen		Negative
Month 7	07/08/2017	No AFB seen		Negative
Month 8	05/09/2017	No AFB seen		Negative

*AFB* acid fast bacilli, *TBC* tuberculosis complex

## Discussion and conclusions

In 2015, the WHO estimated that, 630 (220–1200) people developed MDR-TB in Cameroon among all notified pulmonary TB cases. Children are likely to be exposed to TB in their household or community, especially in endemic areas and the diagnosis of MDR-TB in children is based on a careful history with clinical assessment and follow up [7]. They also have a high risk of progression to active TB and developing severe forms of the disease. The WHO estimates that about 90% of children will develop the disease within 12 months of infection, with immuno suppressed children being particularly vulnerable [5]. Multi-Drug Resistant Tuberculosis in children is mainly the result of transmission of a strain of *M. tuberculosis* from an adult source case [8], and therefore often unsuspected unless a history of contact with an adult pulmonary MDR-TB case is known; as it was the case with our patient; who was a close contact to the mother and having the same bacterial strain.

Unfortunately, several sub-Saharan African countries like Cameroon have not implemented systematic contact tracing for tuberculosis, a key component of disease control, in their routine program mostly due to lack of well-trained human resource and diagnostic constraints.

The WHO recommends focalizing contact tracing on: patients with MDR-TB, smear-positive pulmonary TB, children below 5 years or persons living with HIV and AIDS [9]. The diagnosis of MDR-TB in our patient was the result of a careful contact tracing of his mother performed at the referral treatment center where she was managed.

The knowledge of the Cameroonian population on regular screening of symptomatic TB contacts and control measures of TB infection such as: regular ventilation of the room to reduce concentration of infectious droplet nuclei in the air, personal protective measures with surgical masks and hospital hygiene with sputum containers is poor [7]. Population education on the symptoms of the disease and its prevention would lead to better health-seeking behaviors and progress in eventual control of the disease. This calls for the need to integrate and effectively implement the contact tracing strategy in the national TB program of Cameroon; by training healthcare providers on a systematic screening of all the household and close contact (s) of a pulmonary TB case; taking into consideration the source case strain and assessing the population’s risk factors such as children under five, immuno depressed persons, malnutrition and overcrowding.

This is the first reported case of pediatric MDR-TB in an HIV co-infected child from Cameroon. It was difficult for us to tell if there was a lack of response to antiretroviral therapy (ART) in this child or not. Firstly, the development of MDR-TB in this child is most probably the result of the none response to ARVs with severe immune depression (VL of 23,144 cells/µl; CD4 = 149 cells/µl). Secondly, we could not assess his ART records to evaluate the evolution of his viral counts on treatment. Finally, increase in the viral load and drop in the CD4 cell count could just be due to the fact that the child was severely ill. It was therefore, difficult to say with certainty; there was a lack of response to ART in our patient.

Also, the child’s weight gain was documented as a response measure in the patient treatment form; the weight dropped from 20 kg at treatment initiation to 17 kg at month 3 while increasing to 20.5 kg at the end of treatment in month 9.

The pill burden, drug formulation and related side effects of the MDR-TB treatment are a major obstacle to medication adherence, especially in children [10]. A recent study conducted by Kuaban et al. in Cameroon to assess the outcome of the use of the 9 month short term regimen (4(AmMfxPtoHCfzEZ/5MfxCfzEZ) among second line drug naïve adult MDR-TB patients revealed hearing impairment as the most common adverse drug event [11] compared with the excellent outcomes of the Bangladesh regimen for Multi-drug resistant tuberculosis among over 500 consecutive adult patients [12].

However, Ettehad et al. showed that digestive symptoms such as nausea and vomiting were the commonest side effects in children treated for MDR-TB [2]. Our patient manifested with digestive symptoms (abdominal pains and diarrhea) and bilateral knee pain which we attributed to Moxifloxacin and was managed using oral rehydration solution and anti-inflammatory according to his weight. No major adverse events like hearing loss, psychiatric disorders or hypothyroidism were noted in our patient. Indeed, an audiogram done was normal. Currently, he is being followed up for brownish discoloration of the eyes which could be attributed to Clofazimine following two mechanisms: The drug induced reversible ceroid lipofuscinosis and/or accumulation and recapture of clofazimine in the tissues and organs.

The collaboration between the diagnostic center and the Reference Laboratory was laudable and documented in order to request a second sample for confirmation and archiving the MDR-TB child’s records. During the course of his management, we experienced few side effects which were abdominal pains, joint pains and brownish eye discoloration with no visual impairment for which he is been followed-up at the 9th month. The problems we encountered were the lack of second-line TB drug formulations and pharmacokinetic for children. A shortened regimen enables an earlier return to school and social activities for the child. We therefore advocate the extreme necessity of a systematic contact tracing of each TB patient; and to separate data including children in research investigating the efficacy and safety of a 9 month regimen for MDR-TB; and side effects for second line TB drugs in immunocompromised children.

## Abbreviations

ARVs: anti-retrovirals
DST: drug susceptibility testing
ECG: electro-cardio gram
HAART: highly active anti-retroviral therapy
HCT: hematocrit
HIV: human immune-deficiency virus
MDR-TB: multi-drug resistant tuberculosis
MTB/RIF: mycobacterium tuberculosis/rifampicin
NTP: National Tuberculosis Program
PM-DST: proportionnal microscopic drug susceptibility testing
SDGs: sustainable development goals
SGOT: serum glutamo-oxaloacetic transaminase
SGPT: serum glutamo-pyruvate transferase
TB: tuberculosis
TBC: tuberculosis Complex
WHO: World Health Organization
